# Trends in prevalence, awareness, treatment, and control of hypertension, and the risk of mortality among middle-aged Lithuanian urban population in 1983–2009

**DOI:** 10.1186/1471-2261-12-68

**Published:** 2012-08-31

**Authors:** Regina Reklaitiene, Abdonas Tamosiunas, Dalia Virviciute, Migle Baceviciene, Dalia Luksiene

**Affiliations:** 1 Department of Population Studies, Institute of Cardiology, Academy of Medicine, Lithuanian University of Health Sciences, Sukileliu 17, Kaunas, LT-50140, Lithuanian

**Keywords:** Hypertension, Awareness, Treatment, Control, Risk of mortality

## Abstract

**Background:**

Arterial hypertension (AH) is a main risk factor for the risk from cardiovascular (CVD) and stroke mortality. Only few data was published on prevalence, awareness and management of AH in Lithuania. Development of objective approaches to the treatment and control of AH reduces the risk of mortality. The aim of this study was to evaluate time trends, the prevalence, awareness, treatment and control of AH and risk of mortality among Lithuanian urban population aged 45–64 years during the period of 1983–2009.

**Methods:**

Time trends of AH and risk of mortality were examined in three MONICA health surveys in 1983, 1986, 1992, and in one health survey according to MONICA protocol in 2002 included randomly recruited of 2,218 men and 2,491 women. AH was defined as systolic blood pressure (BP) ≥140 mmHg and/or diastolic BP of ≥90 mmHg or current use of antihypertensive medication. The main outcome measures were all-cause mortality, mortality from CVD, coronary heart disease (CHD) and stroke. The mean duration of follow-up was 11.8 ± 9.2 years. All survey periods were age standardized to the year 2006 of Kaunas population. The estimates of hazard ratio and 95% confidence interval were based on the multivariate Cox proportional hazards regression.

**Results:**

In men during 1983–2002 period hypertension prevalence was 52.1–58.7% and did not significantly change whereas in women decreased from 61.0 to 51.0%. There was a significant increase in hypertension awareness among hypertensive men and women (45.0 to 64.4% and 47.7 to 72.3%, respectively) and in treated hypertensives (55.4 to 68.3% in men and 65.6 to 86.2% in women). Adjusted Cox proportional hazard regression analyses revealed a strong dose–response association between blood-pressure level and all-cause, CVD, CHD and stroke-mortality risk in both men and women groups.

**Conclusion:**

In Lithuanian urban population the prevalence of hypertension remains high. Despite positive changes in hypertension awareness and treatment, hypertension control remains poor. A strong dose–response association between the level of BP and all-cause, CVD, CHD and stroke mortality risk was indicated.

## Background

The review of population-based studies of the prevalence and mortality in Eastern European countries pointed out that the burden of cardiovascular diseases (CVD), coronary heart disease (CHD), and cerebrovascular diseases is high and shows no tendency to decrease in future decades [1]. High blood pressure (BP) is considered to be the leading risk factor for death in the world, causing an estimated 7.5 million deaths per year (13% of all deaths) [2,3]. In 2008, the mortality rates from CHD and from cerebrovascular diseases in Lithuania were 3.6-fold and 2.1-fold higher than those in the European Union countries [1]. The high rates in mortality may be due to sociological, demographic, and epidemiological transitions in populations. Arterial hypertension (AH) is commonly recognized as a risk factor of atherosclerosis, and thus it correlates with the morbidity and mortality from CHD and stroke [4-6]. Control of the major CVD risk factors (AH, dyslipidemias, and smoking) has been widely promoted in order to reduce the risk of CVD and stroke [6,7]. Pharmacological treatment of AH and lifestyle modification have been shown to decrease the risk of CVD complications [7]. However, at first it is very important to know the prevalence of AH in the general population, the levels of awareness, treatment, and control of AH. The main elements of effective control include an improvement in the awareness of AH among both health professionals and the general population – especially among individuals with high BP. Effective treatment and control of AH reduces risk of mortality from CVD and stroke [7]. Detecting AH depends on both the awareness of population and the intervention of medical staff [8]. Epidemiological data have showed that women have a better awareness of AH than do men and their AH is controlled better, but the low rate of control for both sexes calls for further improvements [9,10]. The systematic analysis of health examination surveys and epidemiological studies with 786 country-years and 5.4 million participants [11] informed that global population systolic BP decreased slightly since 1980, but trends varied significantly across regions and countries; systolic BP is currently highest in low-income and middle-income countries. So, the effective population-based and personal interventions should be targeted towards low-income and middle-income countries.

In Lithuania, there are scarce data from rural and urban regions on the prevalence, awareness, treatment, and control AH in a population-based sample [12]. Therefore, the aim of the present study was to estimate the trend and levels of prevalence, awareness, treatment, and control of AH, and to evaluate the risk of AH and mortality among Lithuanian population aged 45–64 years from 1983 to 2009.

## Methods

### Study sample

Data from the four surveys are presented in the article. The firsts, second and the third health examinations in the framework of the Multinational Monitoring of Trends and Determinants in Cardiovascular Disease (MONICA) study [13] were performed in 1983–1984, 1986–1987 and 1992–1993. The fourth survey was conducted in 2001–2002 in accordance with MONICA project protocol. These surveys were carried out in Kaunas city with the population of 348,624. Four random samples of men and women aged 45–64 years, stratified by gender and age, so that at least 200 men and women could be screened in every 10-year age group (45–54, 55–64), were randomly selected from the Kaunas population register. The response rates were for the first survey - 70.2%, for the second – 69.6%, for the third – 58.6%, and for the fourth – 62.4%. Finally, a sample of 4,709 subjects was assigned for statistical analysis. All respondents provided written informed consent. The studies were approved at the regional ethics committee at the Lithuanian University of Health Sciences

### Baseline health examination

In each health survey, the measurements of BP were performed using the same methodology and the information of the awareness and use of drug treatment given were based on the same methods. Arterial BP was measured by the physicians trained in the method using mercury sphygmomanometer and appropriately sized arm cuffs on the right arm. The initial measurement was performed after five minutes of rest on the right arm being at the level of the heart and elbow-assisted. After five minutes, the second measurement was made. The Korotkoff phase 1 (beginning of the sound) and the fifth phase of Korotkoff (disappearance of the sound) was recorded as systolic and diastolic BP. The mean of two readings was used. Definitions and classification of BP levels (mmHg) defined according to the guidelines of the European Society of Cardiology for the management of AH [6]. AH was defined as mean systolic BP ≥140 mmHg and/or mean diastolic BP ≥90 mmHg, and/or antihypertensive drug treatment in the last two weeks. Awareness of AH was determined by patient with AH responding to the question, “Have you ever been told by a doctor or other health care professional that you have high blood pressure?” Treatment of AH was established by participant’s positive answer to the question “Are you currently taking prescribed drugs for high blood pressure in the last 2 weeks?” Control of AH was defined if respondents had been taking drugs for high blood pressure in the last two weeks, and their average systolic BP was of less than 140 mmHg and diastolic BP of less than 90 mmHg. Weight and height were measured with a calibrated scale, and without shoes or heavy clothes. Body mass index (BMI) was calculated as the weight in kilograms divided by the height in meters squared (kg/m^2^). Overweight was defined as BMI from 25.0 to 29.99 kg/m^2^ and obesity as BMI ≥ 30 kg/m^2^. Diabetes mellitus was assessed by a response to a question “Have you ever been told by a doctor that you have diabetes? If, yes, how are you treated? Are you currently taking insulin and/or tablets?”

Smoking habits were assessed according to a current smoking status responding to the question “Do you smoke cigarettes?” A subject who answered “No, I have never smoked” was classified as non-smokers and subjects who answered “No, I smoked in the past but I stopped” was classified as ex-smokers. A subject who smoked at least one cigarette per day was classified as current smoker.

### Follow-up

The participants of the surveys were followed-up from the beginning of each study until December 31, 2009. Mortality data for the period of 1983–2009 were extracted from the regional mortality register. Analysis of all-cause mortality, CVD, CHD and stroke mortality has been performed. The first group consisted of deaths from all causes: 001-E999 – codes of 9^th^ revision of International Classification of Diseases (ICD) (until January 1^st^ 1997); and A00-Z99 – codes of 10^th^ ICD (after January 1^st^ 1997). The second group consisted of deaths from CVD (390–458 – codes of 9th ICD and I00–I99 – codes of 10th ICD). The third group consisted of deaths from CHD (410–414 – codes of 9th ICD and I20–I25 – codes of 10th ICD) and the fourth group consisted of deaths from stroke (430–434 – codes of 9th ICD and I60–I64 – codes of 10th ICD). During 1982–2009, there were 1296 deaths from any cause (807 men and 489 women), 668 deaths from CVD (427 men and 241 women), 408 deaths from CHD (277 men and 131 women), and 139 deaths from stroke (70 men and 69 women). The mean duration of follow-up was 11.8 years.

### Statistical analysis

All surveys were age-adjusted to Kaunas population census of 2006. In 2006, 55% of adults were aged from 45 to 54 years and 45% were 55–64 years of age, with weights ranging between 0.55 and 0.45, which were used in calculating the prevalence. Weights were calculated by dividing the coefficient for each age group by the sum of coefficients for both age groups in each survey. Weighted linear regression to assess time trends from 1983 to 2002 was performed using mean values or percentages and 95% confidence intervals (CI), and p < 0.05 was defined as statistically significant. The estimates of risk ratio and 95% CI were based on the multivariate Cox proportional hazards regression. Data were analyzed with SPSS version 13.4 software for Windows.

## Results

Baseline characteristics of four cohorts aged 45–64 years with AH are presented in Table 1. All the cohorts were homogeneous according to age and gender structure. In CVD risk profiles only few slight positive changes were observed. In women during 19 year period mean systolic BP decreased from 158 (20.3) to 154 (18.9) mmHg (p < 0.05) and mean BMI decreased in men from 32.1 (5.63) to 30.5 (5.36) kg/m^2^ comparing 1983–1984 and 1992–1993 random samples (p < 0.05). Prevalence of the subjects (both men and women) with normal body mass was significantly higher in 1992–1993 as compared to 1983–1984 random sample whereas prevalence of obesity decreased during this period. The proportion of the subjects in 130–139 and/or 85–89 mmHg BP groups increased in men and women populations (p < 0.05). On the contrary, the proportion of the responders in the 140–159 and/or 90–100 mmHg BP group was tend to decrease (p > 0.05).

**Table 1:** Characteristics of population aged 45–64 years with hypertension in 1983–1984 to 2001–2002

	**1983–1984**	**1986–1987**	**1992–1993**	**2001–2002**	**P for trend**
**MEN**	**(n = 450)**	**(n = 313)**	**(n = 241)**	**(n = 252)**	
Mean age, y (SD)	54.4 (5.36)	53.3 (5.31)^1^	54.1 (5.91)	54.0 (55.3)	0.97
Mean BP, mmHg (SD)					
Systolic	154.4 (19.2)	152.9 (18.5)	152.5 (19.1)	152.5 (19.2)	0.25
Diastolic	94.8 (10.3)	96.4 (9.98)	95.4 (10.6)	95.8 (10.1)	0.74
BP, mmHg,% (95%CI)					
<120/<80	0.0 (0-0)	0.3 (-0.3-0.9)	0.4 (-0.4-1.2)	0.0 (0-0)	0.85
120-129 and/or 80-84	0.4 (-0.2-1.0)	0.0 (0-0)	0.4 (-0.4-1.2)	1.6 (0.1-3.1)	0.13
130-139 and/or 85-89	0.4 (-0.2-1.0)	0.3 (-0.3-0.9)	1.2 (-0.2-2.6)	4.0 (1.6-6.4)^1^	0.04
140-159 and/or 90-100	56.0 (51.4-60.6)	57.2 (51.5-62.5)	55.2 (48.7-61.3)	50.8 (44.8-57.2)	0.08
160-179and/or 100-109	27.8 (23.9-32.1)	25.6 (21.1-30.9)	29.9 (24.2-35.8)	31.0 (25.3-36.7)	0.18
> = 180 and/or > =110	15.3 (11.7-18.3)	16.6 (12.8-21.2)	12.9 (8.8-17.2)	12.7 (8.8-17.2)	0.19
Mean BMI, kg/m^2^ (SD)	28.6 (3.72)	29.0 (3.74)	27.9 (4.10)	29.1 (5.25)	0.82
BMI,% (95% CI)					
<25.0 kg/m^2^	15.3 (12.0-18.6)	14.4 (10.5-18.3)	24.1 (18.7-29.5)^1^	17.8 (13.1-22.5)	0.58
25.0-29.9 kg/m^2^	51.4 (46.8-56.0)	45.3 (39.9-50.9)	52.7 (46.4-59.0)	43.5 (37.4-49.6)	0.50
> = 30.0 kg/m^2^	33.3 (29.0-37.6)	40.3 (34.9-45.7)	23.2 (17.9-28.5)^1^	38.7 (32.7-44.7)	0.95
Diabetes,%	2.7 (1.2-4.2)	4.2 (2.0-6.4)	2.5 (0.5-4.5)	4.7 (2.1-7.3)	0.46
**WOMEN**	**(n = 551)**	**(n = 297)**	**(n = 240)**	**(n = 291)**	
Mean age, y (SD)	54.5 (5.28)	54.6 (5.65)	54.4 (5.45)	54.1 (5.43)	0.07
Mean BP, mmHg (SD)					
Systolic	158.0 (20.3)	155.2 (18.2)	156.8 (19.9)	154.0 (18.9)^1^	0.12
Diastolic	94.0 (10.1)	93.7 (10.3)	95.9 (10.2)	92.3 (10.0)	0.61
BP, mmHg,% (95% CI)					
<120/<80	0.0 (0-0)	0.0 (0-0)	0.0 (0-0)	0.7 (-0.3-1.7)	0.12
120-129 and/or 80-84	0.5 (-0.1-1.1)	0.7 (-0.2-1.6)	0.8 (-0.3-1.9)	3.1 (1.1-5.1)	0.08
130-139 and/or 85-89	0.5 (-0.1-1.1)	1.3 (0.0-2.6)	2.1 (0.3-3.9)	6.5 (3.7-9.3)^1^	0.03
140-159 and/or 90-100	55.5 (51.4-59.6)	56.6 (51.0-62.2)	50.0 (43.7-56.3)	47.8 (42.1-53.5)	0.07
160-179and/or 100-109	24.7 (21.1-28.3)	28.3 (23.2-33.4)	30.0 (24.2-35.8)	29.2 (24.0-34.4)	0.30
> = 180 and/or > =110	18.7 (15.4-22.0)	13.1 (9.3-16.9)	17.1 (12.3-21.9)	12.7 (8.9-16.5)	0.43
Mean BMI, kg/m^2^ (SD)	32.1 (5.63)	32.0 (5.56)	30.5 (5.36)^1^	31.8 (6.34)	0.71
BMI,% (95% CI)					
<25.0 kg/m^2^	8.2 (5.9-10.5)	9.1 (5.8-12.4)	17.3 (12.5-22.1)^1^	11.4 (7.7-15.1)	0.57
25.0-29.9 kg/m^2^	28.6 (24.8-32.4)	29.1 (23.9-34.3)	32.1 (26.2-38.0)	26.2 (21.1-31.3)	0.55
> = 30.0 kg/m^2^	63.2 (59.2-67.2)	61.8 (56.1-67.1)	50.6 (44.2-57.0)^1^	62.4 (56.8-68.0)	0.96
Diabetes,% (95% CI)	4.8 (3.0-6.6)	4.7 (2.3-7.1)	2.1 (0.3-3.9)	6.6 (3.7-9.5)	0.65

^1^ P < 0.05, as compared to 1983-1984.

*BP* blood pressure.

*BMI* body mass index.

*CI* confidence interval.

*SD* standard deviation.

Table 2 presents the general prevalence of AH and it’s awareness, treatment, and control in hypertensive subjects during 1983–2002 year period in men and women cohorts. Overall, during 19 year period, the prevalence of AH did not change in men and decreased in women from 61.0 to 51.0%. In men the general rate of AH awareness increased from 45.0 to 64.4% (p for trend 0.03) as well as in woman from 47.7 to 72.3% (p for trend 0.01). In 1983–1984 the proportion of treated hypertensive subjects was only 55.4% in women and 65.6% in men. In 2001–2002 the rate of treated AH increased and again was in favor of women (86.2%) in comparison with men (68.3%). In men AH control was very low and did not significantly change during 19 year period whereas in women increased from 3.5 to 16.6%.

**Table 2:** Prevalence, awareness, treatment, and control of hypertension by sex from 1983-1984 to 2001-2002

	**1983-1984**	**1986-1987**	**1992-1993**	**2001-2002**	**P for trend**
**% (95% confidence interval)**					
Prevalence of AH,%					
Men (n = 2218)	58.2 (54.5-61.5)	52.1 (48.1-56.1)	57.8 (53.1-62.5)	58.7 (54.1-63.3)	0.57
Women (n = 2491)	61.5(57.8-64.2)	50.4 (46.4-54.4)^1^	55.8 (51.1-60.5)	51.0 (46.9-55.1)^1^	0.44
Awareness,%					
Men	45.0 (40.4-49.6)	48.1 (42.6-53.6)	50.4 (44.1-56.7)	64.4 (58.5-70.3)^1^	0.03
Women	47.7 (43.5-51.9)	55.2 (49.5-60.9)	61.4 (55.3-67.5)^1^	72.3 (67.1-77.5)^1^	0.01
Treatment,%					
Men	55.4 (48.5-62.3)	60.3 (52.5-68.1)	63.6 (55.0-72.2)	68.3 (61.2-75.4)	0.03
Women	65.6 (59.8-71.4)	70.7 (63.7-77.7)	84.5 (78.7-90.3)^1^	86.2 (81.5-90.9)^1^	0.11
Control,%					
Men	3.6 (0.1-7.1)	2.2 (-0.8-5.2)	6.5 (1.0-12.0)	12.6 (6.4-18.8)	0.10
Women	3.5 (0.8-6.2)	3.5 (0.1-6.9)	4.8 (1.1-8.5)	16.6 (11.2-22.0)^1^	0.09

^1^P < 0.05 as compared to 1983-1984.

Age, smoking, BMI and diabetes adjusted Cox proportional hazards analyses revealed the respective dose–response associations of BP level with all-cause, CVD, CHD and stroke mortality risk in men and women cohorts (Table 3). Both men and women in BP category 140–159 and/or 90–99 mmHg were only at increased total CVD mortality risk as compared to <140 and <90 mmHg reference category. Despite the number of deaths from stroke was small, stroke mortality risk was also strongly associated with higher BP level: men and women in BP category 160–179 and/or 100–109 mmHg were respectively at 2.68 (1.37–5.22) and 3.68 (1.83–7.41) increased stroke-mortality risk as compared to normal BP category. What is more, BP ≥180 and/or 110 mmHg at the baseline, increased men’s and women’s stroke mortality risk by 3.75 (1.74–8.07) and 5.01 (2.37–10.6), respectively, as compared to BP category <140 and <90 mmHg.

**Table 3:** Prognostic value of variables for the overall mortality, mortality from CVD*, CHD**, and stroke among population aged 45-64 years in 1983-2009

**Variables**	**Overall mortality**	CVD*	CHD**	**Stroke**
	**Relative Risk (95% Confidence Interval)**			
**MEN**	** (N = 807)**	** (N = 427)**	** (N = 277)**	** (N = 70)**
Age, y	1.08 (1.06-1.09)	1.09 (1.07-1.11)	1.10 (1.07-1.12)	1.06 (1.02-1.11)
Blood pressure, mmHg				
<140 and <90	1	1	1	1
140-159 and/or 90-99	1.17 (0.99-1.38)	1.34 (1.06-1.69)	1.17 (0.88-1.55)	1.71 (0.93-3.15)
160-179and/or 100-109	1.36 (1.11-1.66)	1.50 (1.14-1.98)	1.16 (0.82-1.66)	2.68 (1.37-5.22)
> = 180 and/or > =110	2.19 (1.75-2.74)	2.91 (2.17-3.90)	2.46 (1.72-3.54)	3.75 (1.74-8.07)
Smoking				
Never	1	1	1	1
Regular smokers	2.12 (1.80-2.50)	2.08 (1.66-2.61)	2.20 (1.65-2.93)	2.19 (1.24-3.85)
Ex smokers	1.34 (1.12-1.61)	1.37 (1.08-1.73)	1.56 (1.16-2.09)	1.34 (0.74-2.41)
Body mass index, kg/m^2^				
<25	1	1	1	1
25.0-29.9	0.77 (0.65-0.92)	0.96 (0.75-1.24)	0.98 (0.71-1.34)	1.15 (0.59-2.27)
> = 30	0.99 (0.82-1.20)	1.47 (1.12-1.93)	1.59 (1.14-2.22)	1.65 (0.81-3.40)
Diabetes	1.85 (1.33-2.57)	1.78 (1.15-2.75)	2.07 (1.26-3.41)	2.23 (0.80-6.24)
**WOMEN**	** (N = 489)**	** (N = 241)**	** (N = 131)**	** (N = 69)**
Age, y	1.11 (1.09-1.13)	1.16 (1.13-1.19)	1.18 (1.14-1.23)	1.15 (1.09-1.21)
Blood pressure, mmHg				
<140 and <90	1	1	1	1
140-159 and/or 90-99	1.24 (0.99-1.55)	1.42 (1.02-1.98)	1.20 (0.78-1.85)	1.94 (0.98-3.85)
160-179and/or 100-109	1.48 (1.15-1.92)	2.22 (1.55-3.16)	1.75 (1.09-2.81)	3.68 (1.83-7.41)
> = 180 and/or > =110	1.95 (1.47-2.57)	2.56 (1.72-3.79)	1.77 (1.03-3.06)	5.01 (2.37-10.6)
Smoking				
Never	1	1	1	1
Regular smokers	2.12 (1.41-3.18)	2.13 (1.18-3.83)	2.54 (1.17-5.49)	1.08 (0.26-4.44)
Ex smokers	1.09 (0.64-1.86)	1.46 (0.77-2.75)	2.11 (1.02-4.36)	0.49 (0.07-3.52)
Body mass index, kg/m2				
<25	1	1	1	1
25.0-29.9	1.36 (0.99-1.87)	1.42 (0.91-2.20)	1.64 (0.89-3.03)	1.59 (0.69-3.66)
> = 30	1.38 (1.02-1.88)	1.15 (0.75-1.77)	1.27 (0.70-2.33)	1.05 (0.46-2.40)
Diabetes	2.54 (1.83-3.51)	3.63 (2.41-5.47)	5.93 (3.66-9.60)	1.22 (0.38-3.92)

* Cardiovascular disease; ** Coronary heart disease.

## Discussion

AH is a major public health problem because of its high prevalence and strong positive association with CVD and stroke and it is the main modifiable risk factor of CVD [8]. Proportions of hypertensive patients with CVD are very high, but all preventive measures, awareness, treatment and control need to be significantly improved to diminish the prevalence of AH. The main purpose of this study was to determine time trend in the prevalence of AH in 1983–2002, as well as to assess awareness, treatment, and control of this condition among hypertensive men and women aged 45–64 years, and to examine the risk of AH and mortality among middle-aged Lithuanian population. In this survey, the prevalence of AH ranged from 52.1% to 58.7% among men, and from 61.0% to 51.0% - among women in 1983–1984 to 2001–2002, respectively, using 140/90 mmHg as a threshold.

Analysis of data from the 35 countries (systematically review over the past 6 years) show that the prevalence of AH among men and women was lower in developing than in developed countries (among men 32.3% vs. 40.8%; among women 30.5% and 33.0%) [14]. Data from the 24 geographically defined populations of the WHO MONICA Project show, that the age-adjusted prevalence of AH decreased in most and increased in only a few populations: the highest prevalence was found in Turku, Finland, where 60% of men were hypertensive; among women, the highest prevalence of AH was found in Novosibirsk, Russia (54%); the lowest prevalence was found in Catalonia, Spain, among both men (19%) and women (20%) [15]. Data from 6 European countries and national surveys in United States and Canada, show that the prevalence of AH as standard threshold (ie, BP ≥140/90 mmHg or treatment with antihypertensive medication) was highest in Germany (55%), followed by Finland (49%), Spain (47%), England (42%), Sweden (38%), and Italy (38%) [16]. Also the data informed that the prevalence of AH for the European average was 44.2% compared with 27.6% in North America and the prevalence in the United States and Canada were half of the rate in Germany (28% and 27%, respectively) [16]. In France AH (high BP was defined as BP at least 140/90 mmHg and/or taking antihypertensive drugs) is frequent also, particularly in the age group 55–74 years: the prevalence of high BP was greater in men (47%) than in women (35%) and antihypertensive treatment concerned 80% of the hypertensive individuals with most often a combination therapy. Control rates concerned only 38% of women and 22% of men and decreased with age [17]. Data from the National Health and Nutrition Examination Survey (NHANES) 1988–1994 and 1999–2008 show that BP was controlled in an estimated 50.1% of all patients with AH in NHANES 2007–2008, with most of the improvement since 1988 occurring after 1999–2000 [18]. Data from SEPHAR Study (Romania) show that the prevalence of AH was significantly higher in men (50.2%) than in women (41.1%) [19] AH awareness attended significantly lower in men (34.6%) than in women (52.8%); the rate of AH control was 19.9%, with no significant differences between gender [19]. Analysis of data from 35 countries (systematically review over the past 6 years) showed that the mean awareness, treatment and control of AH between developed and developing countries men did not differ (40.6, 29.2 and 9.8%, respectively, in developing countries and 49.2, 29.1 and 10.8%, respectively, in developed countries); among women, the mean awareness, treatment and control of AH were lower in developing than in developed countries (52.7, 40.5, and 16.2%, respectively, in developing countries and 61.7, 40.6 and 17.3%, respectively, in developed countries) [14]. Data from Czech six independent cross-sectional population surveys (the total number of participants was 13,972) show, that since 1985 awareness of AH in Czech population increased in both sexes (men, from 41.4 to 68.4%; women, from 58.9 to 71.4%; both P < 0.001) as did the number of individuals on antihypertensive medication (men, from 21.1 to 58.2%, women: from 38.9 to 58.9%; both P < 0.001). Control of hypertension improved significantly (from 3.9 to 24.6%) over the same period [10].

In our survey as in many other studies [10,14-16,19-23] AH awareness and control were higher in women than in men, but the percentages of control indicate that women had a possibly better perception of hypertension-associated risks. In addition, some positive improvements in AH awareness, treatment and control were observed. According to our results, in men the general rate of AH awareness increased from 45.0 to 64.4% (p for trend 0.03) as well as in woman from 47.7 to 72.3% (p for trend 0.01). In 1983-1984 the proportion of treated hypertensive subjects was only 55.4% in women and 65.6% in men. In 2001-2002 the rate of treated AH increased and again was in favor of women (86.2%) in comparison with men (68.3%). In men AH control was very low and did not significantly change during 19 year period whereas in women increased from 3.5 to 16.6%.

A large number of observational studies have demonstrated that CVD morbidity and mortality bear a continuous relationship with both systolic and diastolic BP [6]. The relationship has been reported to be less steep for coronary events than for stroke which has thus been labeled as the most important AH related complication. In our longitudinal study of four cohorts the dose–response association between BP level and all-cause, CVD, CHD and stroke-mortality risk was observed.

Data from observational study of 12,677 patients, (treated with antihypertensive drugs, without previous congestive heart failure) aged 30–75 years in Sweden (followed for 5 years) show that hazard ratios for CHD and stroke per 10-mmHg increase in updated mean systolic BP in all participants, adjusting for clinical characteristics and traditional risk factors, were 1.08 (1.04–1.13) and 1.20 (1.13–1.27), P < 0.001 [24]. In contrary, the data analysis of 33,372 Japanese men and women aged 40–69 years, free of prior diagnosis of cancer and CVD, stroke incidence show that the stoke incidence was highest for mild AH, and lower for moderate to severe AH in both sexes [4].

Data from the Third National Health and Nutrition Examination Survey and mortality follow-up through 2000 show that CVD mortality risk for uncontrolled AH was 1.74 (95% CI 1.28–2.49, p = 0.007) and for controlled AH 1.15 (95% CI 0.79–1.80, p = 0.53) [25]. BP at a high range of prehypertension (130–139/84–89 mmHg) was associated with increased risk of CVD mortality (hazard ratio 1.41, p < 0.05) relative to BP less than 120/80 mmHg [25]. Epidemiologic data have revealed that the BP control is achieved in only a small percentage of hypertensive patients [10,14-16]. In central European countries, BP control is only 20% to 25% [8].

Data from the Minnesota Heart Survey study show that population mortality trends for stroke paralleled those for AH control and women had lower rates of stroke mortality than did men throughout the period [26]. Data from the Minnesota Heart Survey study show that proportions of hypertensive patients in the aware, treated, and/or controlled categories leveled in the 1980s and 1990s, but improved substantially from 1995 to 1997 and 2000 to 2002 with BP controlled at the less than 140 and/or 90 mmHg criteria in 44% of the men and 55% of the women; and population mortality trends for stroke paralleled those for AH control. Data from the HAHS (Harvard Alumni Health Study) study show that higher BP in early adulthood was associated with elevated risk of all-cause mortality, CVD, and CHD, but not stroke, several decades later [27]. Since the major part of the risk of mortality was due to AH, a population strategy to promote awareness and control of hypertension should be encouraged.

It is important to be aware of several limitations of our results. The present study did not examine a national sample, but rather included only a random sample of 45–64 year-old of urban population. A future study is needed to examine younger and the oldest samples. The analysis of the whole cohort was limited to a single examination that individuals were classified as hypertensive based on measurements that were obtained on a single occasion, although averaged over two readings. During the follow-up, hypertensive subjects without treatment at inclusion may have been treated later, especially since treatment of AH was not getting persistent. Only 12.6% of men and 16.6% of women demonstrated control of AH. Poor control of AH may be attributed to the lack of procedures that increase the level of compliance and medication adherence.

## Conclusions

The results revealed that more than half of Lithuanian men and women living in an urban area and aged 45-64 had AH (≥140 and/or 90 mmHg). Still remains a considerable proportion of hypertensive subjects who were treated (68.3% of men and 86.7% of women), but BP was effectively controlled only for 12.6% men and 16.6% women. Our longitudinal study showed a strong association between the level of BP and risk of all-cause mortality and mortality from CVD, CHD, and stroke. This may be responsible for the absence of a decrease in cardiovascular–related mortality. The lack of AH control in the population as well as the absence of intervention programs for increasing patients’ compliance are issues to be solved by physicians and public health professionals.

## Competing interests

The authors declare that they have no competing interests and non-financial competing interests.

## Authors’ contribution

All authors contributed to the design and the interpretation of the results, and drafted the paper. AT, contributed to the design and commented on the interpretation of the results. DL, and DV, completed statistical analysis. MB, analyzed the data. All authors read and approved the final draft of the paper.

## Pre-publication history

The pre-publication history for this paper can be accessed here:

http://www.biomedcentral.com/1471-2261/12/68/prepub
